# Cognitive Workload and Workload Transitions Elicit Curvilinear Hemodynamics During Spatial Working Memory

**DOI:** 10.3389/fnhum.2019.00405

**Published:** 2019-11-15

**Authors:** Ryan McKendrick, Amanda Harwood

**Affiliations:** ^1^Northrop Grumman - Mission Systems, Falls Church, VA, United States; ^2^Department of Psychology, George Mason University, Fairfax, VA, United States

**Keywords:** fNIRS (functional near infrared spectroscopy), working memory, mental workload transitions, mental workload, cognitive load

## Abstract

Adaptive training and workload management have the potential to drastically change safety and productivity in high-risk fields—including, air-traffic control, missile defense, and nuclear power-plant operations. Quantifying and classifying cognitive load is important for optimal performance. Brain-based metrics have previously been associated with mental workload. Specifically, attenuation of prefrontal activity has been linked to cognitive overload, a cognitive load state associated with degraded task performance. We hypothesized that a similar nonlinearity would be observed for cognitive underload. When underload and overload effects are combined, they should form a cubic function in lateral prefrontal cortex as a function of working memory load. The first of two studies assessed the relationships between spatial working memory load with subjective, behavioral and hemodynamic measures. A cubic function was observed in left dorsolateral prefrontal cortex (LDLPFC; Brodmann’s Area 46) relating working memory load to changes in oxygenated hemoglobin (HbO). The second, two-part study tested the effects of workload transitions to different cognitive load states. Part-one replicated the effects observed in study one and identified transition points for individual performers. Part-two assessed the effects of transitioning to different cognitive load states. Cognitive load state transitions caused a deviation between behavioral measures and induced a significant change in the cubic function relating LDLPFC HbO and working memory load. From these observations, we present a hypothesis associating workload transitions with the disruption of cognitive process integration.

## Introduction

Humans are capable of complex and amazing skills. Skilled performance may be enhanced by adapting training and task constraints to the mental needs of the individual (Chandler and Sweller, 1991). In this context, cognitive load refers to the amount of mental work an individual is doing relative to the amount of mental work an individual is capable of doing (Parasuraman et al., 2008). Cognitive load is correlated with task difficulty—in general, increases in task difficulty often result in increases in cognitive load. However, this aforementioned model does not account for individual differences in ability. Instead of manipulating task demands to create different workload conditions, a more appropriate method to manipulate workload would be to adapt the task relative to each individual’s abilities. For example, if an individual has a maximum working memory capacity (WMC) of 5, a high workload condition may require that individual to hold four items (80% of their maximum) in memory while a low workload task would require the same individual to hold two items (40% of their maximum) in memory. These high and low cognitive load levels would then vary by participant based on their maximum WMC. While the previous example provides a simple view of adaptive task loading, more complex methods and algorithms can be used to better identify the cognitive load state of an individual.

Neuroergonomics techniques allow researchers to assess *in situ* task workload through neurological and behavioral measures. This is accomplished by describing how the brain functions under various cognitive loads. Multiple studies looking at the parametric effects of working memory load show consistent increases in the blood-oxygen-level dependent (BOLD) contrast in dorsolateral prefrontal cortex (DLPFC) and posterior parietal cortical (PPC) regions of the brain (Braver et al., 1997; Cohen et al., 1997; Culham et al., 2001). Increases in oxygenation (Oxygenated–Deoxygenated hemoglobin; HbO-HbR) as measured with functional near infrared spectroscopy (fNIRS) have also been observed during increasing memory load (Ayaz et al., 2012). Examinations of increases in memory load with EEG have shown an increase in frontal midline theta (4 Hz to 7 Hz) power and a decrease in slow (8 Hz to 12 Hz) alpha power (Gevins et al., 1997; Meltzer et al., 2007). fNIRS has also been used to measure the effects of cognitive load in complex tasks. In a supervisory control task where memory load was manipulated *via* the number of aircraft to be supervised, oxygenation in the left DLPFC increased with the number of aircraft (Durantin et al., 2013). Taken together, the evidence suggests that changes in cognitive load can be observed *via* monitoring of lateral prefrontal brain activity when paired with appropriate experimental conditions.

The current literature does provide evidence of a relationship between cognitive load and hemodynamics. However, there is also evidence that the relationship is not always linear. For example, a non-linear trend has been observed during supervisory control tasks (Durantin et al., 2013). In this experiment, individuals navigated remotely operated vehicles (ROVs) through an airspace while avoiding no-fly zones and their cognitive load was manipulated by altering crosswinds, vehicle inertia and memory load regarding supervisory control. Oxygenation had a negative quadratic relationship with increasing demands of vehicle control and memory load in bilateral DLPFC. A strong correlation between increased DLPFC oxygenation in the highest load condition and performance was also observed. This relationship suggests that cognitive load alone does not have a quadratic relationship with functional hemodynamics, but instead supports the attenuation hypothesis, where cognitive overload induces reductions in hemodynamics in the left DLPFC (Durantin et al., 2013). This effect has also been observed during more basic tasks. For example, in a dual-working memory training study, memory load was adapted to one group’s skill acquisition. In the adapted group, a positive quadratic relationship was observed between memory load and total hemoglobin in PFC. However, a different group of participants with their memory load yoked to the adapted group showed a negative quadratic relationship between memory load and total hemoglobin (McKendrick et al., 2014). These findings suggest that the presence of a negative quadratic slope during workload measurement is indicative of cognitive overload.

In real-world tasks, cognitive load transitions occur often, thus the temporal aspect of workload ought to be considered. For example, an air traffic controller must supervise a varying number of planes while their speed, flight trajectories, and even the weather conditions change regularly. It follows that measurement of the effects of temporally dependent and dynamic cognitive load is needed to improve performance prediction. When cognitive load changes from one load level to another, this is referred to as a workload transition. Workload transitions are common in high-risk environments such as aircraft operation, railway operation, nuclear power plant operation, military tank operation, shipping operation, search and rescue, emergency medical services, and operating rooms (Huey and Wickens, 1993; Noel et al., 2005; Yurko et al., 2010).

Further, the direction of workload transitions (i.e., increasing or decreasing) produced mixed evidence regarding their effects on task performance. A number of studies have found that workload transitions negatively impact performance. For example, a seminal investigation tested the effects of incrementally increasing event rate followed by incrementally decreasing event rate in a number of shadowing task. Decreasing event rate produced a decrement in performance (Cumming and Croft, 1973), while studies of an abrupt increase in event rate have shown reduced signal detection performance (Krulewitz et al., 1975). Furthermore, when required to accurately identify the accuracy of a numeric expression, as well as respond as quickly as possible, random changes in task demands increase reaction time and decrease response accuracy (Matthews, 1986). In general, transitioning demands decreases task performance (Krulewitz et al., 1975; Thornton, 1985; Matthews, 1986; Hancock et al., 1995; Cox-Fuenzalida et al., 2004, 2006; Cox-Fuenzalida and Angie, 2005; Cox-Fuenzalida, 2007; Bowers et al., 2014), regardless of the direction of task demand transition. However, it should be kept in mind that, even in the presence of workload transitions, relatively high task demands still result in lower performance relative to lower task demands. Counterintuitively, there is also evidence that a transition to lower task demands induces a greater decrement than a transition to higher task demands (Cox-Fuenzalida et al., 2006) and that the perception of cognitive load transitions effects performance following a transition. Specifically, the higher an individual’s perception of workload at a given time (workload state response) the more sensitive that individual’s performance is to cognitive load transitions (Mracek et al., 2014).

Workload transitions are prevalent in the majority of real-world tasks. However, workload states (i.e., overload, underload, optimal load) and transitions between states are poorly understood. An improved classification of cognitive load states can improve the utility of workload models in explaining and predicting human errors. Improvements in our understanding of transitions between cognitive load states will also improve the application of automated aiding in improving human-machine system performance. We developed two studies aimed at improving our understanding of cognitive load states and the mechanisms underlying the performance effects of workload transitions. Study 1 was designed to develop a hemodynamic model of the cognitive load induced by our memory task. Study 2 was designed to replicate the effects of study 1 and create individualized workload state transitions to develop a hemodynamic model of workload transition.

## Study 1

### Purpose

The purpose of study 1 is 3-fold. The first aim is to develop functions that quantify the effects of spatial working memory load on subjective mental workload, performance, and oxygenated (HbO) and deoxygenated hemoglobin (HbR). We take working memory to refer to a limited capacity store (be it a unique buffer or part of long term memory; Logie, 2011; Baddeley, 2012) that works in conjunction with a cohort of executive functions (Unsworth and Engle, 2007). We chose working memory to study workload transitions because its difficulty can be discretely manipulated where the difficulty of one trial does not inherently affect the difficulty of a subsequent trial. Furthermore, working memory has strong predictive power for basic (Engle, 2002; Unsworth and Engle, 2007) and complex tasks (Endsley, 1995; de Visser et al., 2010; McKendrick et al., 2014). These functions developed through manipulating working memory can be used in follow-up studies on the effects of workload transitions. The second aim of the study is to identify stationary points where the relationship between HbO/HbR and spatial memory load deviates from linearity. Previous work has shown that hemodynamics deviates from linearity when a task becomes mentally overloading (Durantin et al., 2013). The final aim of study 1 is to determine if an underload state of working memory produces a similar deviation from linearity in HbO and HbR as an overloaded state.

### Methods

#### Participants

Thirteen students from a large mid-Atlantic University, aged between 18 and 35 years, with normal or corrected-to-normal vision participated in the study. Participants had no history of neurocognitive disorders. Participants had not taken any substance which affects central nervous system, such as caffeine, nicotine, and alcohol within 3 h of the study. This experiment was approved by the George Mason University Human Subjects Review Board.

#### Materials

##### Spatial Memory Task

**Figure 1:**
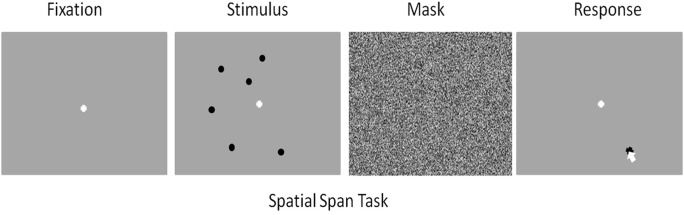
Visual representation of the spatial working memory task. Rest-duration: 8 s, Fixation-duration: 1 s, Stimulus-duration: 1 s, Random Noise Visual Mask-duration: 4 s, Response-duration: variable.

Each trial began with a black screen presented for 8 s, followed by a white fixation cross presented for 1 s. After which the stimuli—randomly spaced black circles (≥ 150 pixels apart) —simultaneously appeared over a gray background for 1 s. Then, a random noise mask was displayed for 4 s across the entire visual field. Finally, another white fixation cross was displayed in the center of a gray screen and participants were required to use the computer mouse to click the locations where the black dots had appeared. Participants were given an unlimited amount of time to respond to the task (see Figure 1). Upon selecting all the locations, the participant would press the space-bar to confirm their response. After the space-bar was pressed the next trial began. Accuracy was defined as the number of circles reported correctly and in the correct location. Inputting more circles than initially presented was penalized. Specifically, if a participant was presented with five circles and input six circles, the number of presented circles was divided by the number of circles reported, the quotient was then multiplied by the number of circles correctly reported. In order for a participant’s response to be recorded as correct they needed to click within a 300-pixel radial distance of the location of the actual circle.

##### The NASA Task Load Index (NASA TLX)

The NASA Task Load Index (NASA TLX; Hart and Staveland, 1988) uses six dimensions to assess task-related workload: mental demand, physical demand, temporal demand, performance, effort, and frustration. Each scale is scored from 0 to 10 is obtained, resulting in a global workload range from 0 to 100. Analyses were performed on the scale for mental demand as previous research has shown this scale has the highest construct validity (McKendrick and Cherry, 2018).

#### Procedure

Participants signed the informed consent, completed a demographic survey, and were fitted with the fNIRS imaging device. The headband has markers on it representing its longitudinal mid-point. These markers were visually aligned with the bridge of the participant’s nose. The bottom of the head band then positioned just above the participant’s eye-brows and adjusted to minimize discomfort and maximize the signal produced. Setup took approximately 15–20 min. Next, participants performed two practice blocks of 10 trials of the spatial memory task. The first practice block presented each possible spatial load level—1 to 10 dots. The second block presented three dots on each trial to acclimate the participant to the experimental design. Then, participants completed 10 blocks with 10 trials per block of each spatial load. The first block had six dots, subsequent trials varied pseudo-randomly. Load order across blocks was set up to minimize correlations with linear and exponential trends. After each block, participants completed the NASA TLX and took a 1-min rest, during which they were asked to close their eyes, to prevent eye strain and minimize the carry over-effects of the differences in cognitive load experience between blocks. Total time for the experiment was approximately 60 min.

#### NIRS Data Acquisition and Processing

**Figure 2:**
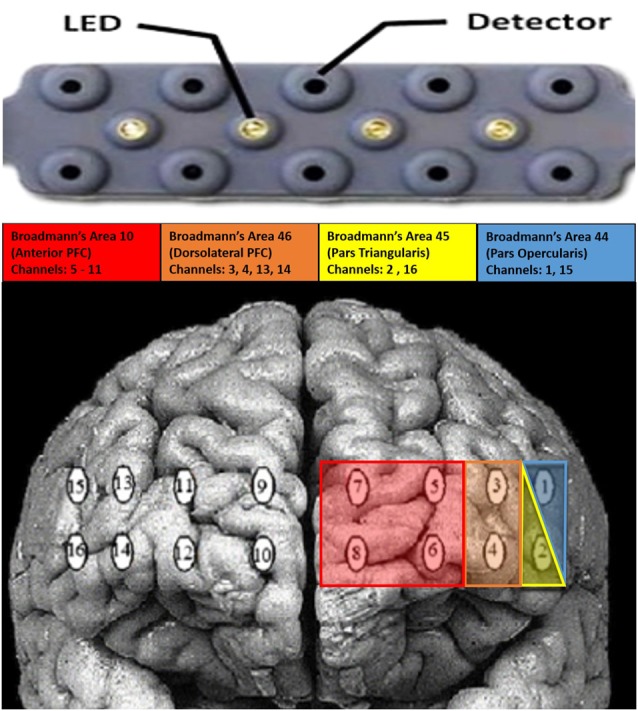
Average projection of the functional near infrared spectroscopy (fNIRS) device over the prefrontal cortex.

Raw light intensities were acquired with an fNIRS Devices fNIR 1000 system with four emitters and 10 detectors placed across the forehead. Headband has five detectors along the top, and five along the bottom of the headband. The four emitters are between the two rows of five detectors. The device used 685 nm and 830 nm wavelengths with a sampling rate of 60 Hz and an emitter to detector distance was 2.5 cm. This configuration produces 16 optical channels or channels. Raw light intensities were: (1) low-pass filtered (cut-off = 0.15 Hz; Ayaz et al., 2011) to remove high-frequency heart rate, blood pressure, and respiration artifacts; and (2) a Sliding-window Motion Artifact Rejection (SMAR) algorithm was used to remove potential motion artifacts (for algorithm details, see Ayaz et al., 2010). Relative chromophore concentrations were calculated by submitting the filtered light intensities to the modified Beer-Lambert law (Path length factor = 1/emitter distance = 1/0.015; Ayaz et al., 2012). These filters and preprocessing steps were conducted automatically using the Cognitive Optical Brain Imaging Studio (COBIStudio; Ayaz, 2005) software provided for use with the fNIR 1000 device. Ambient light through additional wavelengths was measured and found not to adversely affect the fNIRS signal. The approximate cortical projections of the fNIRS device can be seen in Figure 2.

### Results

For each dependent measure (i.e., NASA TLX score, number locations remembered, presence of zero-errors on a trial, and chromophores from optical channels/channels) a linear or logistic (depending on if the response was continuous or binomial) mixed-effects regression was fitted with Bayesian Information Criterion (BIC) to maximize parsimony of random and fixed effects. Fixed effects included linear (y = b + x), quadratic (y = b + x + x^2^) and cubic (y = b + x + x^2^ + x^3^) effects of memory load. Potential models for nested models comparison allowed for intercept (b), linear slope (x), quadratic slope (x^2^) and cubic slopes (x^3^) to vary randomly across individuals. These random effects could include the whole function (i.e., for cubic y = b + x + x^2^ + x^3^), or a specific component such as the quadratic slope only (x^2^). Significant fixed effects pertaining to oxygenated and deoxygenated hemoglobin were submitted to a false discovery rate (FDR) correction procedure to control for multiple comparisons—details of which can be found in McKendrick et al. (2017). These results can be found in “Accuracy of Individual Items,” “Accuracy at the Trial Level,” “Self-Reported Mental Demand,” and “Prefrontal Hemodynamics and Workload” sections.

### Accuracy for Individual Items Within a Trial

The most parsimonious linear mixed-effects model specified a polynomial quadratic fixed effect of working memory load and a random effect of only the quadratic component. The random effect, as expected, implies that individuals differed in terms of the maximum number of dots they could report (i.e., their WMC). However, there was still a parsimonious fixed effect of working memory load. Fixed effect of intercept was non-significant as anticipated (*B* = −0.19, CI = −0.45 to 0.060), but effects of linear working memory load (*B* = 1.23, CI = 1.12–1.34, *p* < 0.001) and quadratic working memory load (*B* = −0.070, CI = −0.080 to −0.060, *p* < 0.001) were significant. Maximal performance corresponded with the report of 5.1 dots at a WM load of 8.9 dots. These values represent estimates of WMC (5.1) and the overload estimate (8.9; OLE).

The fixed effect quadratic relationship is commensurate with the trend expected from a limited capacity relationship. Initially, performance increases as WM load increases, as the capacity limit is reached performance asymptotes. Surprisingly, participants underperformed at loads four and five, both of which are at or below the function estimated capacity limit. On average, when four dots are presented about 3.5 are reported, and when five dots are presented four are reported. Similarly, the performance begins to asymptote at a load of six dots. This trend suggests that information being maintained in working memory begins to degrade above three dots, however, in spite of this, WMC is not limited to three dots. Instead, most likely through compensatory executive processing, capacity can be extended beyond where degradation begins up to about five dots (ranging from 3.6 to 7.5 dots).

#### Analysis of Perfect Trial Accuracy

The most parsimonious generalized linear mixed-effects model among those tested with BIC specified working memory load as a fixed effect and intercept as a random effect. Similar to the random effects observed for number of correct dots reported, in this model random intercepts imply individual differences in WMC. Specifically, in a logistic model (each location response was coded as a binomial response; 1 for correct and 0 for incorrect), the intercept or point of subjective equality (PSE) is the value at which participants have a 50% probability of making no errors, and can be used as an estimate of WMC (Hambleton et al., 1991). Furthermore, the absence of random slopes suggests that an individual’s theoretical cognitive load range did not vary. The steepness of the slope relates to the range of cognitive load as steeper slopes result in a narrower range. Since slope steepness and cognitive load range did not vary, there can be no relationship between an individual’s cognitive load range and their PSE, an estimate of their WMC. The fixed effects are the generalizable estimates of WMC and the rate of transition from strong performance to poor performance. The log-odds of the PSE were 5.62 with 95% CI of 4.82–6.42, *p* < 0.001. The log-odds of slope for working memory load were −0.92, with 95% CI of −1.02 to −0.82, *p* < 0.001. The fixed estimates of 75, 50, and 25 percent probabilities of success occurred at loads of 4.4, 5.6, and 6.8, respectively.

The logistic model was as expected with error-free trials reducing in frequency as memory load increased. Similar to estimates based on the number of correct dots reported, PSE was estimated at 5.6 dots for the logistic model of error-free trials, or half a dot higher than the WMC estimate. While the estimates from the correct location model should be more precise, the logistic model provides estimates of workload range that the other model could not. Specifically, the 75% and 25% estimates were 4.4 and 6.8 dots, respectively. Finally, as in the previous model, error-free trial estimates of WMC and workload range varied across participants, from 3.7 dots with a range of 2.5 to 4.9 to 7.1 dots with a range of 5.9 to 8.3.

#### Self-reported Mental Demand

The most parsimonious linear mixed-effects model specified a linear fixed effect of working memory load and random effects of intercept and working memory load. The random effect implies that individuals differed in terms of their initial impression of the difficulty of the task and the increase in mental demand as working memory load increased. However, after accounting for this individual variance, there was still a parsimonious fixed effect of WM load (*B* = 7.37, CI = 6.45–8.30, *p* < 0.001). As anticipated, mental demand was perceived by participants as increasing linearly with WM load. This supports the conclusion that the increases in task demand both objectively and subjectively increased mental demand.

#### Prefrontal Hemodynamics and Workload

Hemodynamic response scores were calculated for each trial. Following post-processing of the fNIRS signal, group averaged temporal windows for the hemodynamic response were determined across participants and working memory load. Visual inspection of the average trial time series revealed that the peak concentrations of HbO were observed between 6 and 14 s post-stimulus presentation. We selected a temporal window between 6 and 10 s post-stimulus to represent the peak of the hemodynamic response associated with maintenance of the memory stimulus. The time period from 10 to 14 s post-stimulus was not used as it was believed that this period was representative of responding to the stimulus. These conditions are assumed based on the pacing of the trials (0–4 s = stimulus encoding and maintenance; 5-X seconds = active stimulus response), and considering peak hemodynamic activity generally occurs 6 s post-stimulus. Mean hemodynamic response from the defined window was submitted to linear mixed-effects regression on a trial by trial basis for each participant. Analyses were performed on eight optical channels and the channels are labeled hereafter for their approximate anatomical locations based on where they were placed. These eight channels were selected due to their lateral localization and the association between lateral prefrontal cortex and working memory (D’Esposito and Postle, 2015).

Six optical channels had significant parsimonious effects of WM load. These effects were primarily located laterally. The fixed effects for HbO and HbR in each of the six optical channels are presented in Table 1. The model for each optical channel and chromophore were selected independently using BIC measures of parsimony—only models with at least one significant effect are reported here, though all eight lateral channels were analyzed.

**Table 1:** Effects of working memory load on relative concentrations of oxygenated hemoglobin (HbO) frontal cortex for the most parsimonious model.

Channel	Metric	Intercept	WML	WML^2^	WML^3^
Left Pars Opercularis (1)	HbO	0.046 (−0.14, 0.23)	−0.032*** (−0.05, -0.02)	-	-
	HbR	0.007 (−0.20, 0.21)	−0.054 (−0.16, 0.052)	0.0003 (−0.025, 0.025)	0.0004 (−0.001, 0.002)
Left Pars Triangularis (2)	HbO	0.163 (−0.30, 0.63)	-	−0.024** (−0.004, −0.001)	-
	HbR	−0.102 (−0.30, 0.94)	−0.037** (−0.07, −0.01)	0.123 (−0.003, 0.250)	0.003* (0.001, 0.004)
Left Dorsolateral	HbO	−0.039 (−0.22, 0.14)	-	−0.003*** (−0.004, −0.002)	-
Prefrontal Cortex (3)	HbR	−0.097 (−0.22, 0.029)	-	-	-
Left Dorsolateral	HbO	−0.20 (−0.64, 0.25)	0.43** (0.14, 0.71)	−0.116*** (−0.17, −0.06)	0.008*** (0.05, 0.01)
Prefrontal Cortex (4)	HbR	−0.140 (−0.358, 0.077)	0.066 (−0.069, 0.202)	0.032* (−0.061, −0.003)	0.0027** (0.001, 0.005)
Right Dorsolateral	HbO	0.18 (−0.04, 0.40)	-	-	-
Prefrontal Cortex (14)	HbR	0.054 (−0.141, 0.250)	−0.121*** (−0.179, −0.063)	0.0098** (0.003, 0.017)	-
Right Pars Triangularis (16)	HbO	0.74** (0.32, 1.2)	−0.54** (−0.87, −0.2)	0.104** (0.04, 0.17)	−0.006** (−0.009, −0.002)
	HbR	−0.045 (−0.213, 0.123)	0.088 (−0.195, 0.370)	−0.028 (−0.095, 0.040)	0.002 (−0.003, 0.006)

Significant effects are in bold: *indicates < 0.05, **indicates < 0.01, ***indicates < 0.001. Blank values indicate that the effect was not present in the most parsimonious model.

**Figure 3:**
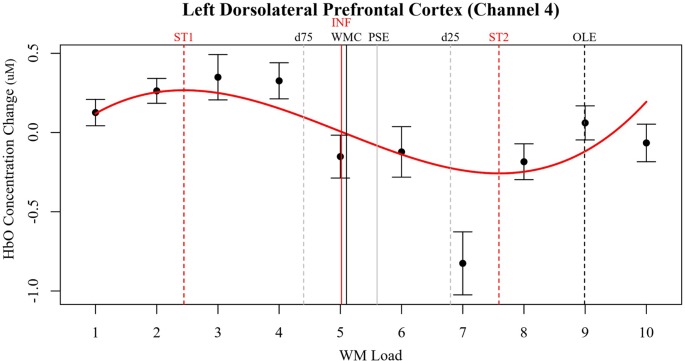
Relative concentration changes in oxygenated hemoglobin (HbO) as a function of working memory load in the LDLPFC (Channel 4). Annotated from the behavioral models are the estimates of working memory capacity (WMC and PSE) as well as bounding estimates of optimal workload (*d*_75_ to *d*_25_). HbO estimates of WMC (INF) and bounding estimates of optimal load are also annotated (ST1, ST2).

The optical channels located approximately over left dorsolateral prefrontal cortex (LDLPFC; Brodmann’s Area 46; Channel 4) and right pars triangularis (RPT; Brodmann’s Area 45; Channel 16) produced the most robust nonlinear cerebral hemodynamics as an effect of WM load (Figure 3). The effects of WM load in LDLPFC and RPT were cubic. In LDLPFC, increased WM load initially increased regional activity. After two to three dots activity decreases, reaching an asymptote at seven to eight dots and increasing again hereafter. There was also meaningful individual variance in the rate of initial increase (in HbO only) and the following decrease in activity (in HbO and HbR). In RPT the effect of WM load on HbO and HbR was different. In RPT increased WM load initially decreased regional activity. After three to four dots activity increases, reaching an asymptote at seven to eight dots and decreasing again hereafter. There was meaningful individual variance in the rate of initial decrease (in HbO and HbR), the following increase in activity (in HbO and HbR) and in the final increase (in HbR only). Of note, in the RPT the fixed effects of WM load on HbR are not significant unlike in the LDLPFC. The lack of significant change in HbR reduces confidence that these effects in RPT are purely due to brain activity. However, the trends in HbR are inverse to those of HbO, so it is possible that these effects were smaller than our threshold for statistical power.

### Discussion

Study 1 aimed to model the most parsimonious relationships between perceived mental demand, behavioral performance, and prefrontal hemodynamics as a function of spatial working memory load. An emphasis was placed on finding parsimonious nonlinear relationships in prefrontal HbO and HbR, with the goal of using components of the nonlinear functions to objectively describe different cognitive workload states at the group and individual level. Exploratory modeling was successful, revealing multiple behavioral estimates of WMC and cognitive state boundaries. Most importantly, two nonlinear cubic polynomial relationships were observed in HbO for optical channels over LDLPFC (Channel 4) and right pars triangularis (Channel 16). These functions can be used in future studies of workload transitions as they both have relatively good coherence with behavioral estimates, expand on those estimates, and measure three different states of cognitive load. Additionally, these workload states could be classified in real-time—which could subsequently be used to adapt individual’s tasking during training and potentially in real work environments.

## Study 2

### Purpose

The purpose of study 2 was 2-fold: (1) replicating the effects of spatial working memory load on behavioral performance, subjective report, and hemodynamics observed in study 1; and (2) testing how transitions between individualized cognitive load states alter performance, self-reported mental demand and prefrontal hemodynamics. Workload transitions in either increasing or decreasing direction have been shown to consistently hinder performance (Cox-Fuenzalida et al., 2004, 2006; Cox-Fuenzalida and Angie, 2005; Cox-Fuenzalida, 2007). However, these studies were not adapted to individuals and showed little to no performance differences. Individually adapted workload transitions are anticipated to additionally tax cognitive resources and hence alter the relationship between prefrontal hemodynamics and cognitive load. The subjective, behavioral and hemodynamic functions observed in study 1 will be used to adapt workload transitions to individuals. Specifically, testing the effects of workload transition direction (up or down) and cognitive load state (under- or over-load) relative to identical cognitive load levels when no transition has occurred by examining changes in the subjective, behavioral, and hemodynamic slope coefficients.

### Methods

#### Participants

Seventeen students from a large mid-Atlantic university, aged between 18 and 45 years, with normal or corrected to normal vision. Participants had no history of neurocognitive disorders. Participants had not taken any substance which affects the central nervous system, such as caffeine, nicotine, and alcohol within 3 h of the study. This experiment was approved by the George Mason University Human Subjects Review Board.

#### Procedure

##### Session 1

Session 1 used the same materials, procedure, and NIRS processing as study 1 for replication purposes. The spatial working memory task can be found in “Spatial Memory Task” section. The NASA-TLX can be found in “NASA TLX” section. The procedure for session 1 can be found in “Procedure” section. NIRS processing for session 1 can be found in “NIRS Data Acquisition and Processing” section.

##### Session 2

Following the break, participants were refitted with the fNIRS imaging device. Participants performed 10 blocks of workload transitions on the spatial memory task. Each transition block was composed of 10 trials, six trials at an initial load level and four trials at the transition level. This distribution of trials was selected to afford a relative balance between pre- and post-transition trials while emphasizing pre-transition. The 10 blocks were composed of six transition conditions and four constant conditions based on the four states of cognitive load. Load order across blocks was set up to minimize correlations with linear and exponential trends. After completing a spatial memory block, participants were asked to report that blocks’ workload *via* NASA TLX. After each block, participants completed the NASA TLX and took a 1-min rest, during which they were asked to close their eyes. Assessment of transition effects lasted for approximately 60 min.

### Results

#### Session 1

The goal of session 1 was 2-fold. First, we aimed to replicate the findings of study one in a new sample. Thus, we repeated the analyses used in study 1 for session 1 of study 2 and compared the beta coefficients and the 95% confidence intervals to assess replication of study 1 effects. These results can be found in “Accuracy of Individual Items,” “Accuracy at the Trial Level,” “Self-Reported Mental Demand,” and “Prefrontal Hemodynamics and Workload” sections. Second, the data from Session 1 was used to identify each participant’s individual task demand levels for the states identified in study 1 to be used in Session 2—this can be found in section “Transition Selection” section.

##### Accuracy of Individual Items

**Figure 4:**
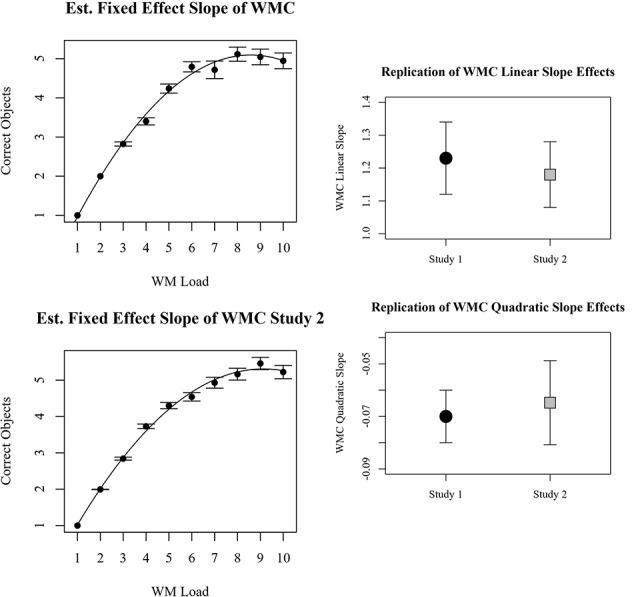
Plots on the left represent behavioral results for the number of objects reported correctly for each memory load. Results from study 1 are top left, results from study 2 are bottom left. Beta coefficients and 95% confidence intervals of linear and quadratic slopes for correctly reported dots in study 1 and study 2 are compared on the right.

The most parsimonious linear mixed-effects model among those tested with BIC specified a polynomial quadratic fixed effect of WM load and a random effect of only the quadratic slope (not the intercept and linear slope). The random effect, as expected, implies that individuals differed in terms of the maximum number of dots they could report. However, after accounting for this individual variance, there was still a parsimonious fixed effect of WM load. Fixed effect of intercept was non-significant as anticipated (*B* = −0.0975, CI = −0.45 to 0.060), but effects of linear working memory load (*B* = 1.18, CI = 1.08–1.28, *p* < 0.001) and quadratic working memory load (*B* = −0.0648, CI = −0.0761 to −0.0534, *p* < 0.001) were significant. From the fixed effects function, it was calculated that maximal performance corresponded with the report of 5.3 dots, and this asymptote occurred at a WM load of 9.3 dots. These values represent estimates of WMC (5.3) and the overload estimate (9.3; OLE). Examination of the beta coefficients and confidence intervals of the effects in study 1 and study 2 reveal that both the linear and quadratic slopes observed in study 1 were successfully replicated in study 2. The comparisons are depicted in Figure 4.

##### Accuracy at the Trial Level

**Figure 5:**
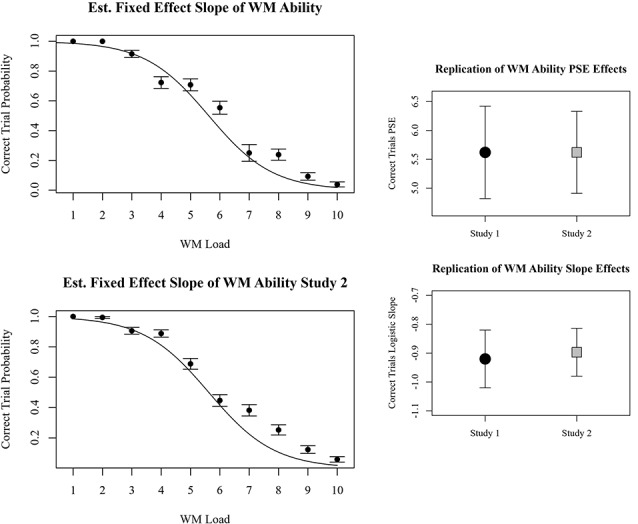
Plots on the left represent behavioral results for perfect accuracy trials for each memory load. Results from study 1 are top left, and results from study 2 are bottom left. Beta coefficients and 95% confidence intervals of linear and quadratic slopes for correctly reported dots in study 1 and study 2 are compared on the right.

The most parsimonious generalized linear mixed-effects model among those tested with BIC specified working memory load as a fixed effect and participant intercept as a random effect. Similar to the random effects observed for number of correct objects reported, in this model random intercepts imply individual differences in WMC. Furthermore, the absence of random slopes suggests that an individual’s theoretical workload range did not vary, and consequentially an individual’s workload range was not related to their WMC. The fixed effects are the generalizable estimates of WMC and the rate of transition from strong performance to poor performance. The log-odds of the PSE were 5.62 with 95% CI of 4.91–6.33, *p* < 0.001. The log-odds of slope for working memory load were −0.897, with 95% CI of −0.980 to −0.814, *p* < 0.001. The fixed estimates of 87, 50, and 13 percent probabilities of success occurred at loads of 3.5, 5.6, and 7.7, respectively. Examination of the beta coefficients and confidence intervals of the effects in study 1 and study 2 reveal that both the PSE and linear slopes observed in study 1 were successfully replicated in study 2. The comparisons are depicted in Figure 5.

##### Self-reported Mental Demand

The most parsimonious linear mixed-effects model among those tested with BIC specified a linear fixed effect of WM load and random effects of intercept and WM load. The random effect implies that individuals differed in terms of their perception of the lowest task difficulty level and the increase in mental demand as WM load increased. However, after accounting for this individual variance, there was still a parsimonious fixed effect of WM load (*B* = 7.15, CI = 5.73–8.56, *p* < 0.001). Examination of the beta coefficients and confidence intervals of the effect in study 1 and study 2 reveal that the linear slope observed in study 1 was successfully replicated in study 2.

##### Prefrontal Hemodynamics and Workload

Hemodynamic response mean values were calculated for each trial. Following post-processing of the NIRS signal, group average temporal windows for peak hemodynamic response were determined by averaging trial time series across participants and working memory load. Visual inspection of the average trial time series revealed that the peak concentrations of HbO were observed between 6 and 14 s post-stimulus presentation. As in study 1, we selected a temporal window between 6 and 10 s post-stimulus to represent the peak of the hemodynamic response for memory load maintenance. The time period from 10 to 14 s post-stimulus was not used as it was believed that this period was representative of responding to the stimulus. Only the channels which showed cubic polynomial effects from study 1 were further analyzed. The most parsimonious models for each channel are reported in Table 2. The channel over LDLPFC (Channel 4) was the only channel to show a significant cubic polynomial relationship with WM load.

**Table 2:** Effects of working memory load on relative concentrations of HbO frontal cortex for the most parsimonious models.

Channel	Metric	Intercept	WML	WML^2^	WML^3^
**Left Dorsolateral Prefrontal Cortex (4)**	**HbO**	1.28 (−0.04, 2.6)	0.46** (0.19, 0.73)	−0.116*** (−0.17, −0.06)	0.008*** (0.05, 0.01)
	**HbR**	0.150 (−0.55, 0.85)	0.43*** (0.23, 0.62)	−0.097*** (−0.14, 0.057)	0.006*** (0.004, 0.009)
**Right Pars Triangularis (16)**	**HbO**	1.97** (0.92, 3.0)	0.20 (−0.09, 0.49)	−0.06 (−0.12, 0.004)	0.004* (0.0008, 0.008)
	**HbR**	1.26*** (0.98, 1.53)	0.253 (−0.30, 0.80)	−0.067 (−0.178, 0.048)	0.005 (−0.044, 0.014)

Significant effects are in bold: *indicates < 0.05, **indicates < 0.01, ***indicates < 0.001.

**Figure 6:**
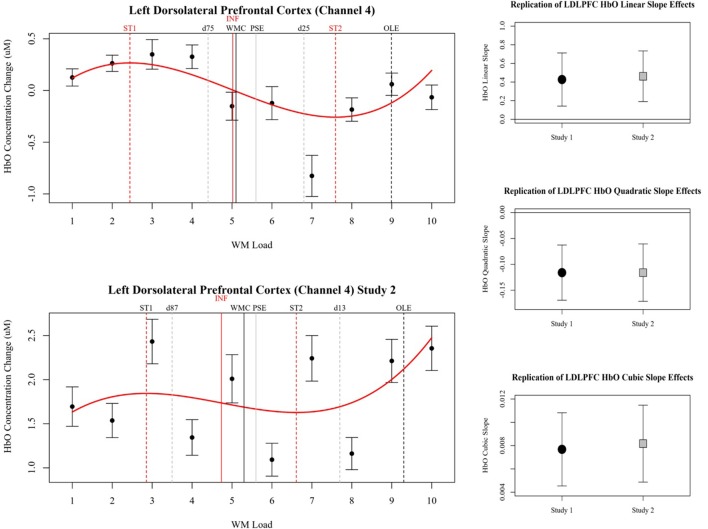
Left plots show relative concentration changes in HbO as a function of working memory load in the LDLPFC (Channel 4). Annotated from the behavioral models are the estimates of working memory capacity (WMC and PSE) as well as bounding estimates of optimal workload (*d*). HbO estimates of WMC (INF) and bounding estimates of optimal load are also annotated (ST1, ST2). Study 1 is depicted in the top left, and study 2 is depicted in the bottom left. On the right, Beta coefficients and 95% confidence intervals of LDLPFC HbO linear, quadratic, and cubic slopes in study 1 and study 2 are compared.

Comparisons of the beta coefficients and confidence intervals of the effects in study 1 and study 2 for LDLPFC are shown in Figure 6. The effects observed in LDLPFC in study 2 successfully replicated the effects observed in this channel in study 1. However, the effects observed in right pars triangularis in study 1 were not replicated in study 2. In study 2 a stronger cubic polynomial relationship was also observed for HbR in LDLPFC, this effect was not present in study 1.

##### Transition Selection

**Figure 7:**
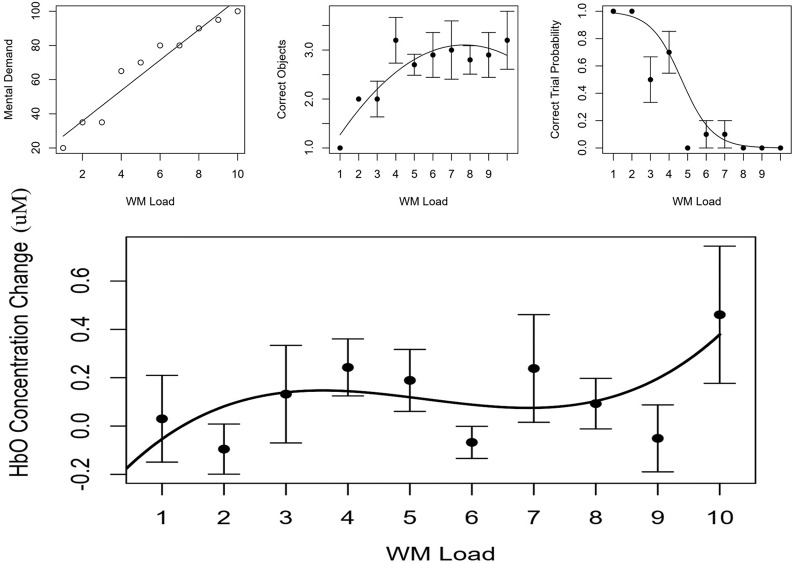
Top Left is participants reported mental demand, top middle is model of correct number of objects reported, top right is probability of perfect accuracy. (Bottom) Relative concentration changes in oxygenated hemoglobin (HbO) as a function of working memory load in the LDLPFC (Channel 4). Annotated from the behavioral models are the estimates of working memory capacity (WMC and PSE) as well as bounding estimates of optimal workload (*d*_75_ to *d*_25_). HbO estimates of WMC (INF) and bounding estimates of optimal load are also annotated (ST1, ST2).

During a 1-h break between session 1 and 2 of study 2, each participant’s subjective, behavioral and hemodynamic data were fit to the functions observed in study 1. Each participant’s WM range was estimated for underload, low load, high load and overload, primarily determined by the stationary points and inflection point observed in each individual’s cubic function for LDLPFC HbO (Channel 4) due to it having parsimonious cubic effects of HbO and HbR in study 1. HbO was used over HbR because it showed a stronger effect in study 1. The presence of multiple stationary points made it easier to define states of overload and underload relative to a quadratic behavioral model. Each estimate of underload and overload transition boundaries was rounded to the nearest whole number, increased and decreased by 1, yielding values both greater and less than the estimates. For extreme cases where participant’s WMC/inflection point was below 4, the minimum (1, 3) was used for underload and low load. When a participant’s inflection point was above 7, the maximum (8, 10) was used for high load and overload. Subjective, behavioral and hemodynamic modeling of a single subject are depicted in Figure 7, while individual parameters are listed in Table 3.

**Table 3:** Individualized participant WM load levels per state.

Participant	Underload	Low Load	High Load	Overload
1	1	3	7	9
2	2	4	8	10
3	2	4	7	9
4	1	3	6	8
5	3	4	8	10
6	1	3	8	10
7	2	4	6	8
8	3	5	6	8
9	1	3	4	6
10	1	3	5	8
11	2	4	6	8
12	2	5	7	10
13	1	3	6	9
14	1	4	7	10
15	1	4	7	10
16	2	5	7	10
17	2	4	5	7

#### Session 2

The goal of session 2 was to compare participant’s performance metric, subjective workload (NASA-TLX), and prefrontal hemodynamics in blocks where there was no workload transition to blocks where there were workload transitions—based on the individualized WM load levels discussed in “Transition Selection” section. Only the last four trials of each experimental block were used in the analyses. Four conditions were created, representing no transitions, transitions, increasing transitions, and decreasing transitions, respectively. The no transition condition was composed of the last four trials for each of the four blocks lacking a transition, yielding estimates for underload, low load, high load, and overload. The transition condition was composed of the last four trials of the six blocks where a transition occurred. The transition blocks were further categorized as increasing, or decreasing task demands in the last four trials—three blocks each. Thus, increasing, decreasing and transition conditions are compared to the no transition condition, not to each other.

For each dependent (subjective, behavioral, and hemodynamic) measure, a linear mixed-effects regression has been fitted with BIC to maximize parsimony of random and fixed effects. Potential random effects were intercept, slope and their combination as either correlated or uncorrelated. These results can be found in “Accuracy of Individual Within a Trial,” “Analysis of Perfect Accuracy,” “Self-reported Mental Demand,” and “Prefrontal Hemodynamics and Workload” sections, respectively.

##### Accuracy of Individual Within a Trial

The most parsimonious linear mixed-effects model specified a polynomial quadratic fixed effect of cognitive load state interacting with workload transitions and random effects of participant intercept and linear slope of cognitive load state. The intercepts for number of correct object on trials with no-transition trials and transition trials were −1.03 (CI = −1.99 to −0.06, *p* < 0.001) and −1.08 (*B* = −0.05, CI = −1.24 to 1.13, *p* > 0.05), respectively. They were significantly different from zero, but not from each other. The linear slopes for on trials with no-transition trials and transition trials were 2.99 (CI = 2.16–3.81, *p* < 0.001) and 2.96 (*B* = −0.03, CI = −1.08 to 1.02, *p* > 0.05, respectively. The quadratic slopes for no-transition trials and transition trials were −0.338 (CI = −0.5 to −0.18, *p* < 0.001) and −0.323 (*B* = 0.015, CI = −0.19 to 0.22, *p* > 0.05), respectively.

##### Analysis of Perfect Accuracy

The most parsimonious generalized linear mixed-effects model specified a logistic slope of workload state interacting with workload transitions and the random effect of participant intercept. The intercepts for PSE on trials with no-transition trials and transition trials were 2.04 (CI = 1.78–2.24, *p* < 0.001) and 1.82 (*B* = −0.223, CI = −0.88 to 0.034, *p* > 0.05), respectively. The intercepts were significantly different from zero, but not from each other. The slopes for on trials with no-transition trials and transition trials were −2.58 (CI = −3.14 to −2.03, *p* < 0.001) and −2.05 (*B* = 0.527, CI = −0.11 to 1.17, *p* > 0.05), respectively. The slopes were significantly different from zero, but not from each other.

##### Self-reported Mental Demand

The most parsimonious linear mixed-effects model specified a linear slope of workload state interacting with increasing and decreasing workload transitions and the random effects of participant intercept and linear slope. The intercepts for mental demand on trials with no-transition trials, increasing transition, and decreasing transition trials were 39.19 (CI = 33.03–43.36, *p* < 0.001), 35.64 (*B* = −3.55, CI = −7.32 to 0.214, *p* > 0.05), and 50.46 (*B* = 11.27, CI = 7.51–15.04, *p* < 0.001), respectively. The intercepts were significantly different from zero. The intercept for decreasing WM load was significantly higher than no transition (*p* < 0.001) but the slope for increasing WM load was not (*p* > 0.05). The slopes for on mental demand on trials with no-transition trials, increasing transition, and decreasing transition trials were 19.33 (CI = 15.42–23.23, *p* < 0.001), 19.71 (*B* = 0.38, CI = −3.36 to 4.13, *p* > 0.05), and 16.62 (*B* = −2.71, CI = −6.45 to 1.04, *p* > 0.05), respectively. The slopes were significantly different from zero, but not from each other.

**Figure 8:**
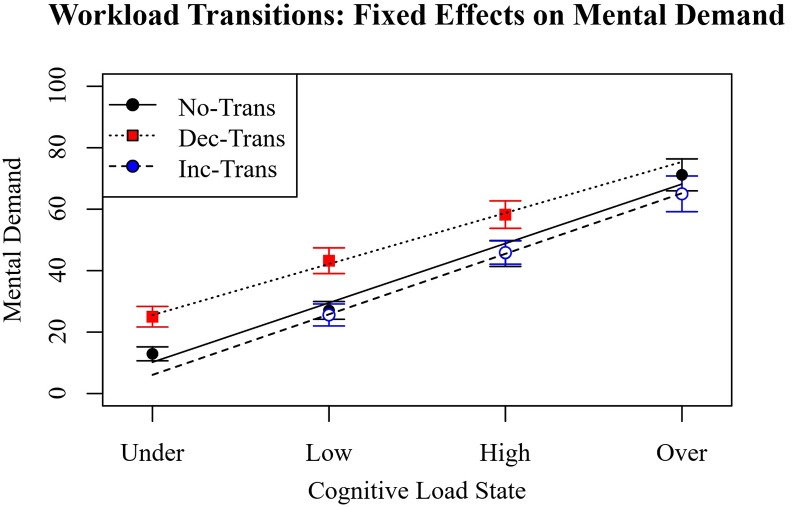
NASA Task Load Index (NASA TLX) mental demand scores comparing the effect of no-transition, transitions of increasing cognitive load, and transitions of decreasing cognitive load across different cognitive load states.

As in previous observations, increasing cognitive load increased the perceived mental demand of the task. As expected, the mean reported mental demand was higher for decreasing workload transitions. This was because during the decreasing condition, participants spent more total time at a higher cognitive load than the no-transitions condition, suggesting an accurate aggregation of the difficulty of the task demands. However, it would then be expected that increasing workload transitions would have a lower mean mental demand since more time was spent at a lower cognitive load. This difference was not observed. The transition comparisons are depicted in Figure 8.

##### Prefrontal Hemodynamics and Workload

**Figure 9:**
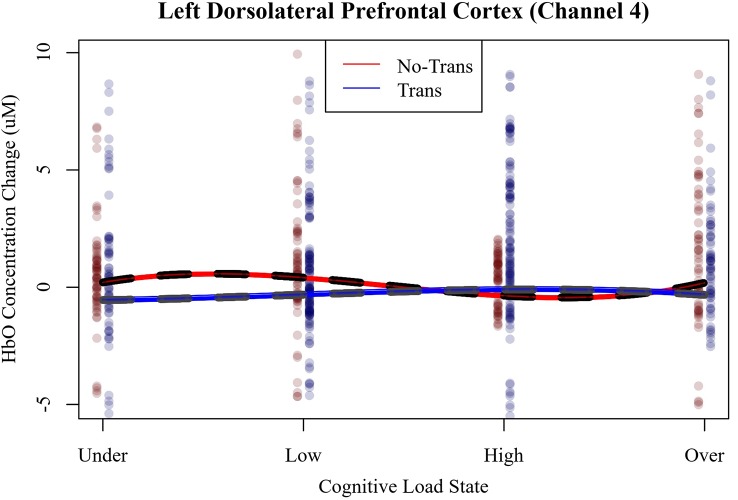
LDLPFC (Channel 4) relative HbO as a function of cognitive load state and workload transition condition.

Since only the effects for the cubic function in LDLPFC (Channel 4) were replicated in study 2, and these effects were primarily used in selecting individual’s cognitive load states, only the hemodynamics of LDLPFC were analyzed for effects of workload transitions. The most parsimonious linear mixed-effects specified a polynomial cubic slope (x + x^2^ + x^3^) of cognitive load state interacting with workload transitions and the random effects of participant intercept and trial slope. The fixed effect slopes of LDLPFC HbO, shown in Figure 9 are plotted across cognitive load states as a function of workload transition condition.

HbO’s cubic function for no-transitions was significant and cohered with the direction of slopes observed in study 1 and session 1 of study 2 in LDLPFC (Channel 4; linear = 5.85, CI = 1.64–10.06, *p* < 0.01; quadratic = −2.77, CI = −4.65 to −0.89, *p* = 0.01; cubic = 0.38, CI = 0.13–0.63, *p* < 0.01). Importantly, no cognitive load states manifested at a stationary point in the HbO function. Instead, as per our design, each cognitive load state manifested either to the left or right of each stationary point. The inflection point in the no transition condition could also be used to distinguish between low and high cognitive load states. The presence of workload transitions significantly altered the shape of the cubic function of HbO in LDLPFC (linear = −0.53, *B* = −6.38, CI = −12.18 to −0.58, *p* < 0.05; quadratic = 0.42, *B* = 3.19, CI = 0.62–5.76, *p* < 0.05; cubic = −0.07, *B* = −0.45, CI = −0.79 to −0.11, *p* < 0.01). Workload transitions effectively flipped the signs of each of the functions’ slopes. It appears as though the presence of workload transitions caused a phase shift in the HbO function. We found no evidence to suggest that workload transitions significantly altered the shape of the HbR function.

### Discussion

Session 1 of study 2 aimed to replicate the effects observed in study 1, providing an empirical foundation from which the effects of workload transitions could be measured. The behavioral and subjective measures from study 1 were successfully replicated in study 2. Hemodynamic effects of HbO were successfully replicated in LDLPFC (Channel 4) but not in RPT (Channel 16).

Session 2 evaluated if workload transitions altered the previously observed relationships between performance, subjective report, prefrontal hemodynamics, and cognitive load. Cognitive load states were classified on an individual basis based on prefrontal hemodynamics and the direction of workload transitions was also manipulated. Comparisons of the effects of workload transitions were made to identical time points at identical load levels where no workload transition had occurred. Behavioral measures of correctly reported objects and correctly performed trials were not strongly affected by workload transitions. Unlike behavioral measures, subjective measures were sensitive to the presence and direction of workload transitions. Specifically, decreasing transitions increased the aggregate perceived mental demand of a trial block, and increasing transitions trended toward indifference from no-transitions. While the behavioral and subjective measures held little insight, the prefrontal hemodynamics observed across cognitive load states were substantially changed by the presence of workload transitions.

The change in prefrontal hemodynamics provides evidence that workload transitions affect cognition, however, the nature of that affect is unclear. While we have provided evidence against additive increases in cognitive load (beyond that induced by task demands) as a sufficient explanation for the affect, we cannot rule out its role entirely. The cubic function we found in LDLPFC (Channel 4) also suggests that there may be another brain region that acts as a moderator to LDLPFC within a greater cognitive load network. The activity of this region is hypothesized (given the observed function in LDLPFC) to coincide with the onset of compensatory cognitive strategies as cognitive load exceeds WMC. Examining the interaction with this yet unidentified brain region could shed further light on the nature of the cognitive changes underlying the hemodynamic changes induced by workload transitions.

## General Conclusion

Optimal human performance and skill acquisition require matching the demands of tasks and training to an individual’s cognitive capacity (Chandler and Sweller, 1991; Parasuraman et al., 2008). In order to adapt tasks and training to individuals, we must be able to classify cognitive load and cognitive states on an individual basis. In the above studies, we examined cognitive load levels and states across subjective, behavioral and hemodynamic measures. In two different random samples and at two different time points in the second sample, we observed consistent effects of spatial memory load on cognitive load across all measures. When the replicability of psychological effects is currently under question (Open Science Collaboration, 2015), the consistency of our observations cannot be understated.

Although our different measurement methods were consistent across samples, their utility was not equivalent. The NASA TLX measure of cognitive load had a linear relationship with spatial memory load. The linearity of this measure meant at best we could differentiate one cognitive load *level* from another. Similarly, behavioral measures could differentiate cognitive load levels; but to a lesser degree due to the nonlinear nature of their effects. The nonlinear properties of the behavioral effects meant they were useful in indexing cognitive ability and minimally a cognitive overload *state*. It was the nonlinearities present in prefrontal hemodynamics that afforded them similar diagnosticity. Prefrontal hemodynamics were also able to index ability and objective measures of cognitive load *states* *via* function differentiation.

The properties observed between cognitive load states indexed *via* hemodynamics can inform system design. The state observed between one and three dots coheres with previous estimates of WMC which is usually estimated at four chunks of information (Conway and Getz, 2010). In system design where WM is highlighted as an *a priori* design consideration, four chunks of information is believed to be the maximal amount of information that can be presented to a user (Wickens et al., 2013). However, we show that when defining mental demand characteristics, task decision biases should be accounted for. In tasks that reward minimal errors, keeping information processing under an individual’s WMC (i.e., one to five objects) will result in maximal performance. However, if a task rewards maximizing hits our results suggest that keeping processing under the capacity limit will not maximize performance. Instead, in these tasks, maximal performance is achieved through providing the maximal information without inducing an overload state (i.e., five to eight objects).

The effects observed in the optical channel over LDLPFC (Channel 4) with increasing cognitive load also alters previous findings regarding the attenuation of prefrontal activity in the presence of cognitive overload. The attenuation hypothesis states that increasing demand to the point of cognitive overload attenuated the response to cognitive load in prefrontal cortex (Durantin et al., 2013). In some sense, our observations still cohere with this hypothesis. Indeed, attenuation does occur in prefrontal hemodynamics as errors begin to arise, however, these are only errors in regards to flawless performance. They do not represent overload in the traditional sense. Overload results in a total breakdown in task performance (Grier et al., 2008). This did not occur in our data until about eight objects, where performance across all measures is in decline. This also coincided with increasing hemodynamic activity.

In light of expanding on the attenuation hypothesis, we should also endeavor to understand why we did not observe a state of underload, and if there are yet to be observed cognitive states beyond the state we classify as cognitive overload. The state of underload is notorious for being difficult to measure and in future work cognitive state classification and workload transition tests may need to be adapted to a task that has the correct properties to induce this important cognitive state. At the same time, previous work assumed an attenuation hypothesis for cognitive overload only because the range of task demands was too narrow. This begs the question as to whether there are other cognitive states related to increased task demands that could not be observed in the current paradigm. It is possible that extending the range of observed task demands could alter the model observed here.

Our success in objectively identifying cognitive load states allowed us to test the effects of workload transitions. Workload transition effects have been consistently observed in subjective measures of workload but we observed minimal effects. Yet, increasing workload transitions did show evidence of biasing of subjective report to the cognitive load experienced during the workload transition. There are two ways of interpreting this evidence. Either, there are different subjective effects for increasing and decreasing workload transitions, or there is no particular effect of either and instead, it was overall task demands that biased subjective report. More specifically, individuals during no-transitions, increasing transitions and decreasing transitions only reported their perceived cognitive load for the highest task demands experienced on a given block. Given the simplicity of the second explanation, it is a more favorable option.

Similar to our observations regarding subjective measures of cognitive load, we did not observe the strong effects we expected in behavioral performance. There is a substantial body of research showing the effects of workload transitions. The prominent effect is a decrease in performance, and this is independent of whether the transition is to a higher or lower cognitive load (Cox-Fuenzalida et al., 2006). Here, workload transitions had little to no effect on an individual’s ability to maintain and recall spatial working memory objects. However, there was a trend for workload transitions decreasing the probability of flawless recall. Yet, this is a more nuanced effect than what we expected to observe. It is unlikely that the failure to replicate behavioral decrements from workload transitions is due to the transition tests occurring in the second session. When we examine performance across sessions 1 and 2, there is little change in performance. This lack of change overtime was expected as improvements in this task require either extensive training or non-invasive brain stimulation (McKendrick et al., 2014, 2015). There was also no evidence of fatigue, which has been observed in previous behavioral and hemodynamic measurements during this task. We also feel that expectancy of the transition had little effect on the outcome. Previous work in this area has shown that the knowledge of whether a workload transition will or will not occur does not change the workload transition’s effect on behavior (Goldberg and Stewart, 1980). We also do not believe that the workload transitions we employed were too subtle. Each transition was to a markedly different level of performance, and transitions subtler than those employed here have still successfully altered performance (Cox-Fuenzalida, 2007).

Our paradigm did deviate from traditional workload transition paradigms in two ways, and either one of these deviations could have altered the effect of workload transitions on performance. First, we altered cognitive load *via* altering memory load. The vast majority of previous workload transition studies have used stimulus event rate to alter cognitive load (Cumming and Croft, 1973; Krulewitz et al., 1975; Goldberg and Stewart, 1980; Hancock et al., 1995; Cox-Fuenzalida et al., 2004, 2006; Cox-Fuenzalida and Angie, 2005; Cox-Fuenzalida, 2007; Bowers et al., 2014). Second, we adapted workload transitions to the cognitive states of each individual. There are few instances where (Afergan et al., 2014; Bowers et al., 2014), workload transition studies have made an attempt to adapt task demand manipulations to individuals. Without adaptation, individual differences in cognitive capacity can result in different levels of cognitive load in different individuals even when task demands are identical.

It is possible that workload transitions disrupt the integration of cognitive processes involved in working memory. Due to this disruption, individuals default to a simpler model of the task with a greater emphasis on satisficing (i.e., just good enough performance). We find evidence of this disruption in the deviation between an individual’s ability to correctly report objects and their ability to perform flawlessly. A disruption of process integration could explain why complete task execution declines with no observable change in total objects reported. This trend could explain individual’s upward bias in self-reported workload. Evidence of disruption *via* workload transitions can also be seen in prefrontal hemodynamics. In the presence of workload transitions, attenuation occurs at much higher task demands compared to trials where no transition occurred. Attenuation could be occurring at a higher cognitive state because the mechanisms for integrating compensatory executive functions with primary task performance are operating less efficiently. It is possible that the other brain regions that moderate the function in the LDLPFC optical channel (Channel 4) and compensatory functions require a greater level of activation following workload transitions. Future research should directly investigate this theory of disruption.

We can make two other explicit predictions regarding workload transitions if we assume that they cause a disruption in cognitive process integration. First, tasks composed of a greater number of cognitive components, or more complex responses should be more negatively affected by workload transitions. This runs counter to our initial assumption that research on workload transitions should focus primarily on basic tasks, and also explains why workload transitions are of such a concern in real work. Second, the disruption hypothesis suggests that workload transitions should have specific network effects. Effectively, brain regions associated with the onset of attenuated activity in lateral prefrontal cortex as cognitive load increases should have their functional relationship retarded following workload transitions. The observation of these two effects would provide direct evidence for the hypothesis that workload transitions cause a disruption of cognitive process integration.

## Limitations

One of the most notable limitations of our results is associated with study 2 where an individual’s transitions were adapted in relation to baseline data collected earlier that day. We cannot say for certain if our methods would produce similar results if the experiment spanned across multiple days or months. However, as WMC is conceptualized as a trait-level characteristic (Baddeley, 2012)—rather than state-dependent—we are optimistic regarding future research investigating multi-day transfer. Regarding signal cleaning of the fNIRS data, our system did not contain short channel measurements that have been used to remove aspects of physiological noise from the fNIRS signal. Considering, that continuous-wave fNIRS is baseline corrected, and our analyses should have aided in correcting for certain time-dependent physiological changes, it seems unlikely that our results are attributable to physiological noise. However, future studies should leverage short channel measurements to provide another robust method of removing physiological noise. Additionally, we would be remiss if we did not mention that we used a convenience sample composed primarily of undergraduate students enrolled in Psychology courses. The use of a convenience sample leads to a *potential* generalizability issue. However, since the spatial working memory task required no expertise or domain-specific knowledge, we believe our results have high generalizability.

## Data Availability Statement

The datasets generated for this study are available on request to the corresponding author.

## Ethics Statement

This study was carried out in accordance with the recommendations of the Belmont Report and George Mason University Internal Review Board with written informed consent from all subjects. All subjects gave written informed consent in accordance with the Declaration of Helsinki. The protocol was approved by the George Mason University Internal Review Board.

## Author Contributions

Tasks were designed and data analysis was completed by RM. Data collection was completed and the final manuscript was prepared by RM and AH.

## Conflict of Interest

RM and AH were employed by Northrop Grumman during the preparation of the manuscript. The authors declare that this study received funding from Northrop Grumman. The study was designed, and data was collected, analyzed and interpreted prior to the author’s employment at Northrop Grumman. Northrop Grumman was not involved in the study design, data collection, analysis and interpretation of data, the writing of this article or the decision to submit it for publication.
